# Strategies to Improve Adherence to Eye Care Referrals for Children following Vision Screening: A Scoping Review

**DOI:** 10.21203/rs.3.rs-7436894/v1

**Published:** 2025-10-22

**Authors:** Afua O. Asare, Amy Amoah, Patrice M. Hicks, Allison M. Howard, Teasha Luu, Aurora Rodriguez

**Affiliations:** John Moran Eye Center, University of Utah; University of South Florida Morsani College of Medicine; University of South Florida Health Libraries; University of South Florida Health Libraries; Spencer Fox Eccles School of Medicine; Oregon Health & Science University

**Keywords:** Vision Screening, Referral and Consultation, Eye Care Services, Health Services Accessibility, Health Literacy, Health Behavior, Health Services Needs and Demand, Health Services Research, Child, Socioeconomic Factors, Health Disparities, Delivery of Health Care, Access to Health Care, Scoping Review, Public Health, Medically Underserved Area, Vision health literacy, Follow-up care, Barriers to care, Patient navigation, School-based vision screening, Health Knowledge, Attitudes, Practice, Health Education, Intervention Studies, Community Health Services, Preventive Health Services

## Abstract

**Background:**

Vision screening plays a critical role in identifying potential vision disorders; however, its effectiveness is compromised when follow-up with eye care professionals is not completed. Up to 60% of children with abnormal vision screenings do not attend recommended appointments with eye care providers. This study aims to identify and synthesize the strategies to improve adherence to eye care referrals following abnormal vision screening tests.

**Methods:**

The Joanna Briggs Institute methodology was followed to conduct the scoping review. This review is part of a broader study exploring adherence to pediatric eye care referrals. A companion scoping review focusing on social risk factors of non-adherence has been submitted separately. Searches for relevant literature were performed across bibliographic databases and gray literature from their inception to July 2023, with an updated search in November 2024. The review protocol was registered with Open Science Framework. Four reviewers screened and extracted data from the included studies. Strategies were synthesized, and themes developed.

**Results:**

Sixteen studies assessed strategies to overcome social risk factors. The most frequently reported strategy was enhanced direct communication such as phone calls and mailed notifications (n = 13), and logistical support, including transportation and pre-scheduled appointments (n = 9).

**Conclusion:**

A variety of strategies have been implemented to improve adherence to pediatric eye care referrals, with communication and logistical support being the most common strategies used. Significant gaps, however, remain in evaluating the effectiveness of these strategies, especially in low-income countries.

## Introduction

To prevent long-term visual impairment, the early detection and treatment of vision disorders during childhood is essential.(1–3) Vision screening enables the early identification of vision disorders through referrals to eye care professionals for a comprehensive eye examination.(4) However, the effectiveness of vision screening programs is compromised when referrals to eye care professionals are not completed, preventing timely treatment for identified vision disorders. Vision screening is conducted in elementary schools, pediatric primary care, and/or other community-based settings in most countries.(1, 5)

In spite of the widespread use and established benefits of vision screening and comprehensive eye examinations, ensuring follow-up eye care after abnormal screening results remains a significant challenge. Studies show that as many as 60% of children do not attend recommended appointments with eye care providers following abnormal vision screening tests.(2, 3, 6, 7) Lower adherence to eye care appointments disproportionally affects children from socially disadvantaged populations, including racial and ethnic minorities, underserved rural communities, people with low socioeconomic status, and sexual and gender minorities.(4, 8–11)

Efforts to address low adherence to eye care appointments include developing evidence-based strategies and interventions. A previous study reviewed strategies to improve adherence to eye care professionals for adult populations.(12) Strategies reported in the study included patient navigation and prescheduled appointments. It is however unknown if these strategies have been used in pediatric populations and their impact on referral adherence for children.(12) These gaps highlight the need for a comprehensive synthesis of strategies for improving referral nonadherence in children. The objective of this study is to identify strategies from the existing literature to improve referral adherence for comprehensive eye exams by eye care professionals following abnormal vision screening tests for children.

## Methods

### Protocol and registration

This scoping review followed the methodology outlined by the Joanna Briggs Institute (JBI) (6) for scoping reviews and adhered to the Preferred Reporting Items for Systematic Reviews and Meta-Analyses extension for Scoping Reviews (PRISMA-ScR) guidelines.(7) This review is part of a broader study exploring adherence to pediatric eye care referrals. A companion scoping review focusing on social risk factors of non-adherence has been submitted separately, with adaptations to address a distinct research objective focused on intervention strategies. Searches were conducted across bibliographic databases (PubMed, Embase, CINAHL, PsycINFO, Scopus, and Web of Science) and gray literature were searched from their inception to July 2023, with an updated search performed in November 2024. The protocol for this review was registered with the Open Science Framework. As this study involved analysis of previously published sources with de-identified data and did not involve interaction with human participants, ethical approval and informed consent were not required and was exempt from the University of Utah Institutional Review Board.

### Search Strategy

The review team met with a librarian (AMH) in June 2023 to develop a comprehensive search strategy for PubMed. The strategy was subsequently adapted for use across additional electronic databases. The initial literature search was conducted in July 2023 covering studies available from the inception of the respective databases up to July 2023. An updated search was conducted in early November 2024. The search aimed to identify relevant studies that examined social risk factors of and strategies to improve referral adherence following abnormal vision screenings in children. Search terms included keyword and controlled vocabulary, where applicable, and their synonyms for the following concepts: *child, vision screening, referral adherence, and eye care professional*. The complete search strategy used for PubMed can be found in Appendix I.

### Eligibility criteria

Studies were selected based on specific characteristics including the type of target population, outcomes and study design. Screening for eligibility criteria was conducted in two phases: an initial review of the title/abstract followed by a full-text assessment, as detailed in Table 1. Eligible studies included vision screening conducted in any setting, e.g., school, pediatric primary care, or another community-based setting. There were no limitations on date of publication or country of publication; however, studies not published in English were excluded (Table 1).

### Information sources

Searches were conducted across multiple electronic databases, including PubMed (NLM), Embase (Elsevier), CINAHL (EBSCO), PsycINFO (EBSCO), Scopus (Elsevier), and Web of Science (Clarivate). Sources of grey literature were OAIster, NIH RePORTER, Trials Register of Promoting Health Interventions (TRoPHI), National Center for Children’s Vision and Eye Health website, American Association for Pediatric Ophthalmology and Strabismus website, Conference Proceedings Citation Index, and Cochrane CENTRAL Register of Controlled Trials. Additionally, a review of citations in included studies and relevant reviews was performed.

### Evidence selection

Search results were initially collected in EndNote for deduplication and then uploaded into the Rayyan-Intelligent Systematic Review program (Rayyan Systems Inc., Cambridge, MA) to review retrieved articles. The article selection process followed a two-step approach: a review of titles and abstracts, followed by a review of full-text articles. Eligibility was assessed according to the criteria described earlier. All reviewers (AOA, PMH, AA, and AR) were involved in a pilot test on a random 10% of full-text articles retrieved from the initial search to ensure inter-rater reliability (IRR). The reviewer team screened the articles using the initial eligibility criteria outlined in our protocol and made minor changes to the inclusion criteria, such as the addition of studies involving caregivers of children, and clarity on the type of vision screening programs. Screening began once at least 75% agreement had occurred between all four reviewers on the pilot test. Each study was independently assessed at both stages by two reviewers at each level (title, abstract, and full-article review). Discrepancies were resolved through discussion or by a third reviewer (AOA or PMH).

### Study selection and data collection process

Data from studies that were included in the review were extracted using a pilot-tested form. Key variables collected included author, year of publication, geographical context (i.e., city/state, country), World Health Organization income level, target population, screening setting, age range, and sex/gender, race, and ethnicity distributions (Appendix II)

### Synthesis of findings

Data extracted from full-text studies selected for inclusion were synthesized following a three-stage process: inductive coding, theme generation, and theme mapping. Inductive coding was conducted using descriptive coding labels created inductively by one reviewer (AA) to allow patterns and preliminary subthemes related to social risk factors and strategies to emerge from existing published knowledge. The codes were reviewed and confirmed by a second reviewer (AOA) to ensure accuracy.

Theme generation involved the identification, analysis, and interpretation of patterns within the data set (‘thematic synthesis’). Coded data was organized in Microsoft Excel (Microsoft Corporation, Redmond, WA, USA) by one reviewer (AA) into thematic groups, which were subsequently reviewed by another reviewer (AOA) to confirm consistency. The approach of theme generation facilitated the synthesis of findings to extend beyond the original studies, offering analytic insights. During the generation of themes, the reviewers consistently considered the study’s overarching research question and objectives.

## Results

**Figure 1:**
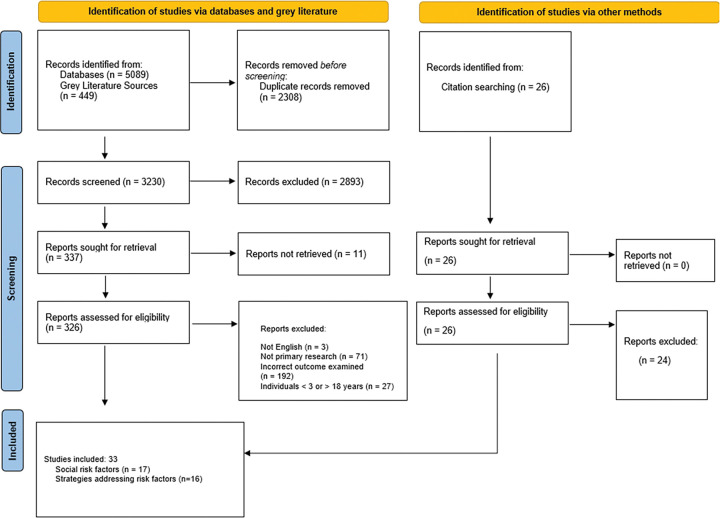
PRISMA Diagram

The initial search identified 5,538 studies through databases and grey literature, with 26 additional records identified through citation searching. After 2,308 duplicates were excluded, 3,230 studies were screened at the level of the abstract and title. An additional 2,893 citations were removed in the title and abstract screening as not relevant. During the full-text screening stage, 337 articles were required for retrieval, of which 11 were not available for retrieval. Of the 326 full-text articles assessed for eligibility, 31 studies met the inclusion criteria and were included in the review. Citations of included studies were searched to identify any additional studies, and two were subsequently included, making a total of 33 included studies. Overall, 16 studies assessed strategies to improve rates of referral adherence (Fig. 1). Seventeen studies assessed social risk factors following an abnormal vision screening test and have been reported in a separate manuscript.

Of the included studies that assessed strategies to overcome referral nonadherence, 14 (88%) studies targeted vision screening programs conducted in schools (15–28), and two (13%) studies targeted programs in health care settings. (29, 30)

**Figure 2:**
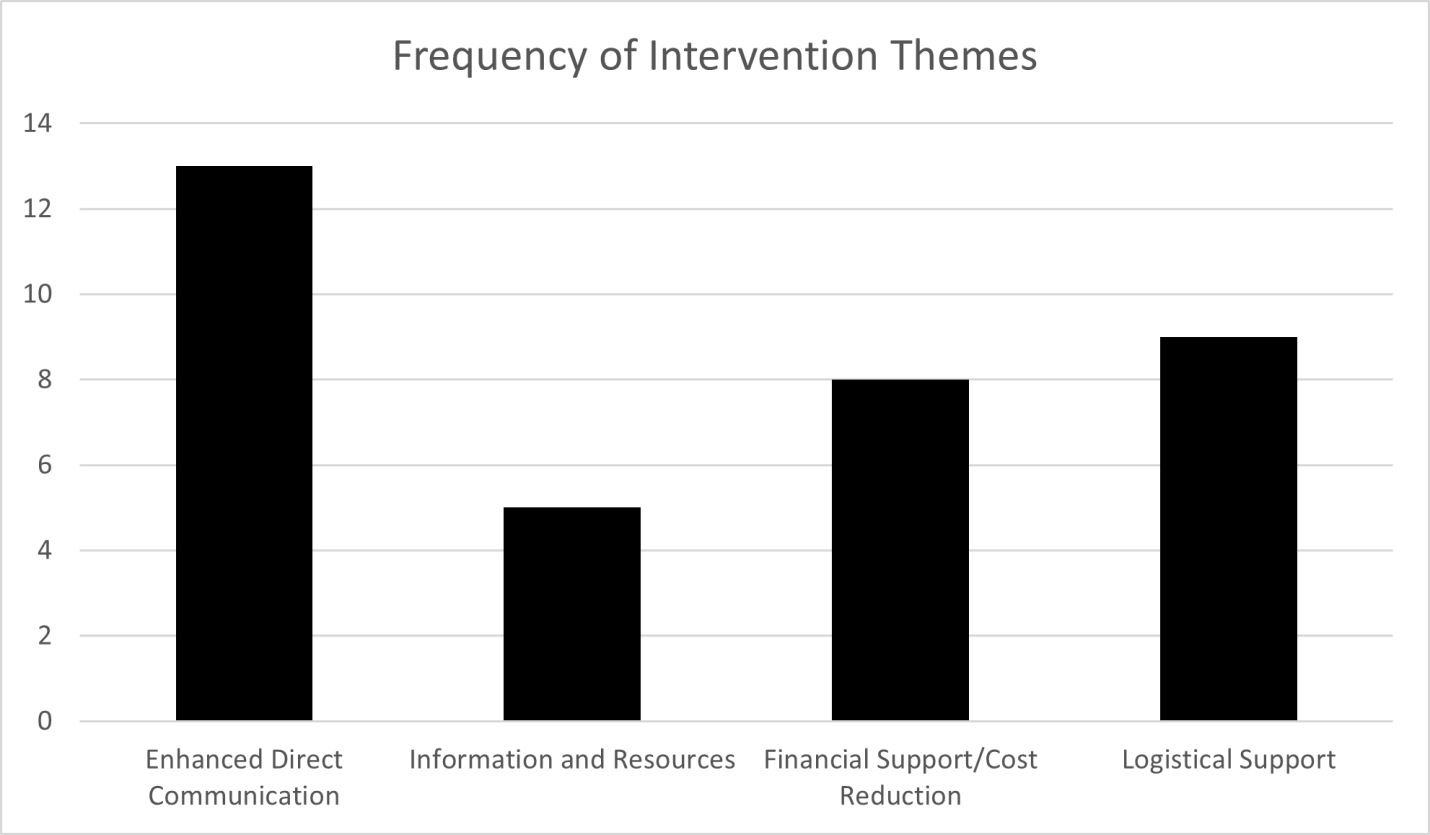
Frequency of Themes Addressed by Papers Included in the Scoping Review

Vision screening programs in two studies (13%) targeted preschool-aged children (2 to 5 years) [16]; four studies (25%) targeted school-aged children (6 to 19 years) [22, 23, 25, 26]; and nine studies (56%) targeted both preschool- and school-aged children (2 to 19 years).(15, 17, 20, 21, 24, 27, 28, 30, 31). Race was reported in three (19%) studies, (22, 26, 30) and ethnicity was reported in four (25%) studies.(16, 17, 22, 24) Nine studies targeted underserved or socially disadvantaged populations.(15, 16, 18, 22, 24, 25, 28, 29, 31) Study designs varied with 10 included studies being intervention (63%) (15, 16, 18, 20–22, 24, 25, 30, 31) compared to three descriptive (19%) (17, 23, 28) designs to assess strategies to improve referral adherence. Retrospective design was used in two (13%) studies (32, 33), and one (6%) study implemented a cluster-randomized controlled trial. (27) In total, 12 studies (75%) were conducted in high income countries [United States (US) (15–18, 21, 22, 24–26, 29–31)], and four (25%) studies were conducted in an upper middle-income country [(Peru, (20) Brazil, (23) China (27, 28)]. The most frequently reported strategy for improving referral adherence was enhanced direct communication reported in 13 studies (81%) (Fig. 2).(16, 17, 21–31). This was followed by logistical support (15, 17, 18, 20, 24, 27–29, 31) in nine (56%) studies and financial support (15, 16, 18, 20, 21, 28, 31, 33) in eight studies (50%). The least reported strategy was related to information and resources (16, 20, 21, 25, 27) in five studies (31%).

### Direct Communication

Enhanced direct communication was implemented in vision screening programs reported by 13 studies (81%), (16, 17, 21–31) that were conducted in upper-middle [Brazil, (23) China (27, 28)] and high-income countries [United States (US), (16, 17, 21, 22, 24, 25, 30–33)]. Particularly, organizers of vision screening programs in nine studies reported the placement of phone calls to caregivers to notify them of referrals after a vision screening test and encourage adherence. Another popular strategy reported in five studies was helping caregivers to schedule referral appointments (e.g., answering caregivers’ questions about covering the cost of the follow-up appointment, type of eye care provider to see, and where to find these providers), and coordinating these appointments. In some cases, pre-arranged appointments with eye care professionals were provided. (Table 3)

### Information and Resources

Five studies (31%) conducted in high [US (16, 21, 25)], and upper-middle income [Peru,(20) China (27)] countries reported the provision of additional information and resources to motivate caregivers to adhere to referrals, and simplify the process of referral adherence. The resources and information included lists of pediatric eye care professionals, the insurance the provider accepted, and contact information to facilitate appointment scheduling. (16, 21) Suchoff et al. created opportunities for optometrists to meet one-on-one with caregivers of children identified with severe vision impairment to amplify the importance of adhering to the referral. (25) A vision screening program in China offered comprehensive eye examinations for referred children, delivered by local nurses, ocular technicians, and ophthalmologists. These providers were trained by specialists from the Zhongshan Ophthalmic Center, a renowned eye clinic affiliated with Sun Yat-sen University. To promote caregiver adherence to referrals, program organizers emphasized the training received by these providers from Zhongshan experts, who would also be present at the referral clinic to oversee the management of any diagnosed eye conditions.(27) (Table 3)

### Financial Support

Vision screening programs reported in eight studies (50%) provided financial support (15, 16, 18, 20, 21, 28, 31, 33) to improve adherence to referral eye exams. The vision screening programs were conducted in high-income (US) (15, 16, 18, 21, 31, 33) and upper-middle-income [China, (27) Peru (20)] countries. To overcome the financial constraints related to referral eye exams for families, the most popular strategy of financial support was free comprehensive eye exams in the same location or within proximity to the vision screening. (15, 18, 28) A study conducted in rural Peru trained teachers to perform basic vision screening of students. (20) Children with abnormal vision screening tests were examined onsite by an optometrist at school. Adherence rates were not reported, but a high follow-up rate with onsite eye exams was implied. (20) Other strategies to provide financial support include the use of social workers to assist families in enrolling in affordable health insurance programs, (20, 33) vouchers for transportation to referral appointments, (20, 33) and financial subsidies for glasses and treatment of eye disorders. (20) A novel approach by Hark et al. was to provide two free movie tickets to caregivers who adhered to referral appointments with eye care professionals for their children. (31) (Table 3)

### Logistical Support

Logistical support was used to improve adherence in nine studies (56%) from upper-middle [China, (27, 28) Peru (20)] and high-income countries [US, (15, 17, 18, 24, 31, 33)]. The most frequently used method was assistance with scheduling and coordinating referral eye appointments to include providing pre-arranged appointments with eye care professionals. (17, 24, 27, 31, 33) Also mentioned in five studies was the provision of comprehensive eye exams on the same site as (or within close proximity to) the vision screening to encourage adherence to referral eye appointments. (17, 24, 27, 31, 33) In addition to this, support for transportation (vouchers and covering cost of transportation), (20, 33) and reserved slots at eye clinics to expedite referral appointments had been strategies employed in prior studies. (31)

### Rates of Referral Adherence

In total, 10 studies (63%) reported rates of adherence pre- and post-strategy intervention, (18, 20–24, 27, 28, 30, 33) while four studies reported rates of adherence post-intervention only. (16, 25, 26, 31) The highest rates of adherence were 96% (an increase from 67% pre-intervention) in the US-based study by Rodriguez et al., followed by 92% (an increase from 82% pre-intervention) in the Peru-based study by Latorre-Arteaga et al, and 91% in the study by Yawn et al. (26) The lowest rate of adherence of 43% in a study by Zhang et al. conducted in China. (28) Rodriguez et al. and Latorre-Arteaga et al. used multiple strategies to improve rates of referral adherence. Specifically, Rodriguez et al. used direct communication (i.e., phone calls to notify and encourage adherence to follow-up appointments) and logistical support (i.e., assistance with scheduling and coordinating follow-up appointments) strategies. (24) Latorre-Arteaga used information and resources (i.e., written health education materials for caregivers), and financial/logistical support (i.e., social workers’ assistance with enrolling in health insurance, transport vouchers, and subsidies for treatment and glasses). (20) Yawn et al., on the other hand, used one communication strategy, i.e. phone calls to notify and encourage adherence to follow-up appointments. (26) In the screening program conducted in China reported by Zhang et al., the lowest rate of referral adherence (43%) was reported despite the use of multiple strategies to improve referral adherence, specifically, financial/logistical support (i.e., free comprehensive eye exams and prescription glasses within proximity of vision screening) and communication strategies (i.e., phone calls to notify and encourage follow-up eye exams).

The highest increase in the rates of adherence to referrals post-intervention was 57% reported in the study by Dotan et al., (33) followed by a 40% increase in adherence in the study by Kripke et al., (30) and a 37% increase in the study by Neville et al. (22) The vision screening programs described in these studies employed different strategies to improve rates of referral non-adherence. (22, 30, 33) For instance, in the study by Dotan et al., they used communication, financial, and logistical support strategies, specifically, social workers, transportation vouchers, and the collection of multiple contact numbers for the caregivers of children who were referred. (33) In the study by Kripke et al., they used a single communication strategy, specifically, sending letters to parents and family physicians to inform them of the referral and need for a comprehensive eye exam. (30) The program in the study by Neville et al. also used two communication strategies; specifically, follow-up phone calls to caregivers and a detailed informative letter (separate from the standard referral notification). (22)

## Discussion

This scoping review synthesized published evidence on strategies designed to increase adherence to pediatric eye care referrals following abnormal vision screening results. Across the 16 included studies, (13–28) direct communication with caregivers emerged as the most frequently implemented approach to improve referral adherence, (14, 15, 18, 19, 21, 22, 24–30) often combined with logistical or financial supports to address common barriers.

### Gaps in the Literature

None of the included studies reported strategies to improve referral adherence in lower-middle and low-income countries. This lack of studies highlights a significant gap in our understanding of the availability and types of strategies in settings where resources may be limited despite the high burden of vision impairment. (31) The limited research in eye care topics in lower-middle and low-income countries. (32–34) may reflect the overall lower investment in global health research. (35–37)

### Free Comprehensive Eye Exams

Free comprehensive eye exams were frequently offered as a means of financial and logistical support to improve referral adherence. However, the impact of free comprehensive eye exams on referral adherence was mixed. In the study by Chu et al., which provided on-site free comprehensive eye exams in low-income schools by an optometrist, only 52% of children returned consent forms, and adherence rates were reported as similar to traditional referrals. (13) The Wills on Wheels Mobile Eye Unit provided follow-up eye exams by pediatric ophthalmologists at school for children referred by an optometrist with positive feedback from school nurses. (16) The presence of the mobile eye unit led to a statistically significant improvement in follow-up rate from a historical rate of 53% to an improved rate of 62%. However, in rural China, vision centers established to offer free eye exams and a pair of glasses for children while increasing access to comprehensive eye exams, resulted in only 43% of referred students visiting vision centers.(16)

### Impact of Strategies on Referral Adherence

Strategies implemented had varying impact on adherence to referrals for children with the rates of adherence ranging from 43–96%. The highest increase in adherence pre and post implementation of a new strategy was 57%. The rate of adherence did not appear to be additive based on the number of strategies employed. That is, employing more than one strategy did not translate to higher rates of adherence. While communication strategies were the most frequently used, it remains unclear whether it had greater impact on rates of adherence compared to other strategies. Improvements in the rates of adherence may depend on the target population and confounding factors such as race and ethnicity, geography, and socioeconomic status. Causal inferences of the effect of strategies on adherence to referrals cannot be drawn using scoping review methodology. To draw inferences and therefore assess the effectiveness of strategies on referral adherence, alternative methodologies such as systematic reviews, meta-analyses or randomized controlled trials are needed.

### Comparisons to Prior Studies

Our companion study indicated that the most common social risk factor for referral nonadherence is a lack of affordability and awareness. Therefore, the results of this current study reflect that of the prior, given that communication, financial, and logistical support were the most frequently reported strategies. The study results also closely reflect that of Zeng et al. who reported health interventions to increase follow-up rates for ophthalmology eye exams following vision screening in the community. (12) The study population in the study by Zeng et al. was adult patients with diabetic retinopathy, glaucoma, refractive error, or cataract, and the most frequently reported interventions were free or subsidized follow-up eye exams (financial support), and reminder phone calls (logistical support). Other interventions identified in the Zeng et al., study included prescheduled ophthalmology appointments (logistical support), transportation assistance (logistical/financial support), patient education (communication), and patient navigators (logistical support). Rates of adherence ranged between 38 and 83%. The highest follow-up rate of 83% was reported in a study by Hark et al., who used patient navigators (logistical support) to improve follow-up to ophthalmology appointments.(38) Patient navigators have been used widely in various medical specialties, including ophthalmology in adult populations (38–42) with no evidence of their effectiveness for vision screening programs in pediatric populations. Future studies should explore the effectiveness of the abundance of health interventions, including patient navigators, to improve referral adherence in vision screening programs targeted specifically to children.

### Strengths and Limitations

Strengths of this study include its novelty, and its robust and systematic approach. To the best of our knowledge, it is the first study to identify strategies to improve referral adherence for children following abnormal vision screening tests, regardless of the screening setting or disease focus. Despite its strengths, the limitations of this study are consistent with JBI methodology and need to be considered. First, the quality of included studies was not assessed. Consequently, the risk of bias within individual studies cannot be commented on, which may affect the strength of the evidence synthesized. Also, had an assessment of the quality of included studies been conducted, additional gaps in knowledge may have been elucidated. Similarly, we cannot assess the effectiveness of strategies identified in prior studies without employing a systematic review or meta-analysis. Also, the heterogeneity of included studies, combined with the exclusion of non-English literature, may have led to gaps in the evidence captured. Finally, despite using a robust and systematic approach to search the literature, it is possible that some relevant studies may not have been selected for inclusion.

## Conclusion

Many strategies—most commonly direct caregiver communication—have been implemented to enhance referral adherence among children flagged by vision screening programs. Future work should rigorously evaluate the effectiveness of these strategies using randomized controlled trials or in systematic reviews and meta-analysis. This knowledge will inform the development of scalable, evidence-based solutions that can prevent avoidable childhood vision loss.

## Figures and Tables

**Table 1:** Inclusion and Exclusion Criteria

	Inclusion	Exclusion
Population	• Parents or guardians of children requiring follow-up after vision screening • Children aged 3 to 18 years at the time of their vision screening test	• Exclusion: Children < 2 years and > 18 years
Study Outcomes	• Patient social risk factors of referral nonadherence to eye care professionals following abnormal vision screenings in children and strategies to address them • Strategies to improve referral nonadherence to eye care professionals following abnormal vision screenings in children and strategies to address them	• None
Type of study	• Primary peer-reviewed studies, regardless of design	• Commentaries, letters to the editor, editorials, and review papers

**Table 2:** Sociodemographic Characteristics of Target Populations in Included Studies

First author, Year Published	Geographical Context	WHO Income Level	Target Population	Screening setting	Age range**, years	Sex/gender distribution [Females/Girls n (%)]	Race	Ethnicity
Chu et al., 2015	US, Santa Ana, California	High Income	Parents of at-risk students attending Title I schools*	Title I school	-	NA	NA	NA
Clarke et al., 2008	US, Southern California	High Income	Parents of preschool children in a large, urban public-school system	Preschool	2 to 5	Parents: not specified Children: 292 (48.7%)	NA	Latino/Hispanic 516 (86%)
Day et al., 2022	US, New York City	High Income	Parents of students attending public school	School	3 to 18	Children: 129,035 (48.6%)	Hispanic: 114, 696 (43.2%); Black: 62,603 (23.6%); Asian: 42,785 (16.1%); White: 42,141 (15.9%); Multiracial or Missing: 3,287 (1.2%)	Hispanic:114,696 (43.2%) Non-Hispanic: 150,816 (56.8%)
Diao et al., 2016	US, Philadelphia, Pennsylvania	High Income	At-risk/socioeconomically disadvantaged elementary students	Elementary school***	-	NA	NA	NA
Dotan et al., 2015	US, Philadelphia, Pennsylvania	High Income	Parents of children with low SES	Wills Eye Hospital	0 to18	Parents: not specified Children: 471 (51%)	NA	NA
Hark et al., 2016	US, Philadelphia, Pennsylvania	High Income	Parents of children from underserved communities	Elementary school	5 to 12	Children: 5,245 (49%)	NA	NA
Kripke et al., 1970	US, Iowa	High Income	Parents of preschool children	Field clinics	4 to 6	NA	NA	NA
Latorre-Arteaga et al., 2016	Peru, Abancay, Apurimac region	Upper Middle Income	Preschool, primary, and secondary school children; teachers and directors	Schools	3 to 17	NA	NA	NA
Musch et al., 2020	USA, Wayne County, Michigan	High Income	Parents of children in grades 1 and 3	schools	5 to 10	78 (48.1%)	NA	NA
Neville et al., 2015	USA, New Jersey	High Income	Parents of school-aged children in an urban middle school	School (urban middle school)	11 to 14	47 (54.0%)	African American 36(41.4%), Caucasian 23 (26.5%), Multiracial 3 (3.4%), Asian 3 (3.4%)	Hispanic 22 (25.3%)
Noma et al., 2012	Brazil, Guarulhos, Sao Paulo	Upper Middle	Public elementary school children in grade 1 to 4	Schools	7 to 10	5540 (50.70%) screened	NA	NA
Rodriguez et al., 2018	USA, San Jose, California	High Income	School nurses and parents of minority public school children with low income	Public schools	5 to 13	NA	NA	Latino/Hispanic students in demonstration schools 81.1%, and 70.2% in comparison schools
Suchoff and Mozlin, 1991	USA, Bronx, New York	High Income	Parents of inner-city high school students (62% Hispanic, 27% Black)	School	14 to 19	Not specified 47 (54.0%)	NA	NA
Yawn et al., 1996	USA, Rochester, Olmstead County, Minnesota	High Income	Parents of kindergarten children in public and private schools	Public and private schools	-	NA	97% White	NA
Zeng et al., 2020	China, Yudu county, Jiangxi province	Upper Middle Income - Parts of China have been categorized as High Income	Parents of preschool children with abnormal screening results	Preschool	4 to 7	535 girls (46.4%), 254 (42%) Control & 241 (50%) Intervention Group	NA	NA
Zhang et al., 2021	China, Shaanxi and Gansu Provinces	Upper Middle Income - Parts of China have been categorized as High Income	Children in grade 4 to 6 living in poor, rural counties	School	9 to 12	7,648 (48.5%) of children with visual impairment, 2718 (52.6%) of those complying with referral	NA	NA

NOTES

* Title I school = publicly funded school with a large portion of the surrounding attendance area are low-income families

** Refers to age range of children in the study sample

*** Elementary school with more than 80% of students falling below the Federal Poverty Line (household income < $19,090 for family of three or < $23,050 for family of four)

ABBREVIATIONS - US: United States of America; NA: Not Applicable – study did not provide

**Table 2:** Identified Barriers and Interventions to Improve Referral Adherence to Eye Care Professionals

First author, Year published	Country and local context	Participants	Barriers to Referral Adherence	Adherence (%) pre-strategy	Interventions to Improve Referral Compliance	Outcomes	Adherence (%) post- strategy
Chu et al., 2015	US, Santa Ana, California	1,306 screened, 382 (29%) with abnormal screenings	1) Unable to afford cost of care 2) Need to travel to an ECP	NA	1) Provided on-site eye exams via grant provided by KVLF at no cost to families with free glasses 2) In the event that the consent form was not returned, school personnel attempted to call the student’s parent/guardian	52% (198/382) of parents consented to comprehensive eye exams	NA
Clarke et al., 2008	US, Southern California	592 screened, 42 (7%) with abnormal screenings, 29 (69.0%) answered follow-up calls	NA	NA	1) Provided brochures to families following abnormal vision screenings detailing recommendations and low-cost/no-cost vision care options and the school nurse’s phone number for assistance 2) School nurses conducted follow-up calls to check in and emphasize importance of follow up if patient had not followed up within 2–3 months after an abnormal vision screening result	- 17% (5/29) plans to follow-up after receiving brochure - 83% (20/24) agreed to obtain a vision exam after follow-up calls - 76% (19/25) contacted to obtain results of follow-up exam. - 79% (15/19) with known follow up care	- 17% (5/29) plans to follow-up after receiving brochure - 83% (20/24) agreed to obtain a vision exam after follow-up calls
Day et al., 2022	US, New York City	84% (n=229,834) of Pre-K to 1^st^ grade students enrolled from 2018–19 were screened; Of the total number of students screened, 22.2% (n=42,859) of Pre-K to 1^st^ grade students had abnormal vision screenings, 69.1% (n=29,615) received follow-up efforts	1) Difficulties accessing eye exam services	NA	1) SVP conducts follow-up calls immediately after screening, alerting parents to referral letters placed in the children’s backpack 2) Subsequent phone calls every two weeks to encourage and help parents find pediatric eye care. If family unreachable, letter mailed to parents	Of the 22.2% (n=42,827) of screened students with abnormal vision screenings in 2018–19, 69.1% (n=29,598) received follow-up efforts, and 38.8% completed eye exams (n=16,617)	NA
Diao et al., 2016	US, Philadelphia, Pennsylvania	132 referred, 72% (95/132) students with consent to receive care by the WOW Mobile Eye Unit	1) Lack of transportation	53%	WOW Mobile Eye Unit*	62% (CI: 54% to 70%) of patients (n= 82/132) were seen by the mobile unit. Historic rate of 53%. Statistically significant improvement in follow-up was noted (p = .036)	62% (n=82/132)
Dotan et al., 2015	US, Philadelphia, Pennsylvania	924 children examined, 10% (n=96) needed follow-up care.	1) Lack of insurance or lack of awareness about how to use insurance/insurance benefits 2) Challenges with literacy 3) Inconvenience of follow-up 4) Lack of understanding about the benefits of early intervention 5) Family priorities 6) Concerns about costs 7) Transportation issues 8) Lack of effective communication methods	2%	1) Social worker was present during screening to ensure sufficient and accurate contact information was collected from parents (up to 5 telephone numbers were recorded for children needing follow-up) 2) Social worker helped arrange follow-up appointments, enrollment in insurance if needed, and vouchers for transportation to follow-up when needed	59% (n=57/96) received follow-up care	59% (n=57/96)
Hark et al., 2016	US, Philadelphia, Pennsylvania	10,726 screened, 5% (n=509) referred because of suspected nonrefractive eye disease, 215 scheduled for follow-up appointment, 82% (n=177/215) did not have an eye care provider.	NA	NA	1) Social worker contacted parents and assisted with scheduling an appointment with pediatric ophthalmology at Wills Eye Hospital, which offered expedited appointments in reserved slots 2) Two movie tickets to incentivize parents to attend the follow-up appointment 3) Liaison with the school district conducted up to 3 phone call reminders to parents who did not return completed consent forms within 4 weeks	Referral adherence was 72% (n=127/177)	72% (n=127/177)
Kripke et al., 1970	US, Iowa	1233 screened (585 in 1967, 648 in 1968) 92 referred (49 in 1967, 43 in 1968)	NA	22.4% (n=11/49)	1) Parents and physicians encouraged to arrange full eye examinations for student with abnormal vision screening tests 2) Shortened time between abnormal vision screening and sending report letters to parents 3) Forms requesting replies were enclosed with report letters to parents	63% (n=27/43) of children referred in 1968 completed an eye examination vs 22.4% (n=11) in 1967	62.8% (n=27/43)
Latorre-Arteaga et al., 2016	Peru, Abancay, Apurimac region	1,522 screened, 259 (17%) referred	NA	66% (n=45/68)	1) Develop health education materials for families 2) Provide logistic support (i.e., transportation) 3) Provide outreach clinic in remote locations, free eye exams, and financial subsidies for patients who could not afford the cost of glasses, medical treatment or transport to the eye hospital	237 received an eye exam representing a 39% increase in attendance rate at eye clinic [from 66% (45/68) to 92% (237/259)]	92% (n=237/259)
Musch et al., 2020	USA, Wayne County, Michigan	162 with abnormal screening tests	NA	48% (n=39/82)	1) Coordinator contacted parents within 3 weeks of mailing the initial letter to check if the follow-up examination has been scheduled or was completed 2) Coordinator provided reminders and a list of ECPs in the area willing to examine the child, including designation of those who accept Medicaid as well as those who would see the child at no cost 3) If no completed ECP form was received within 6 weeks of abnormal screening, coordinator made a second phone call to ensure follow-up care is being pursued	65% (52/80) children had a documented ECP exam within 16 weeks in the enhanced group vs 47.6% (39/82) in the standard group (p=0.025)	65% (n=52/80)
Neville et al., 2015	USA, New Jersey	341 screened, 87 abnormal screens	1) Didn’t receive or lost referral letter 2) Financial concerns 3) Lack of insurance 4) Language barriers 5) Lack of time/busy/procrastination 5) Do not believe vision screening results 6) Inattention to school documentation	17% (n=17/100)	Within 2 weeks of mailing an informative letter, school nurses conducted follow-up telephone calls	84% (71/85) either returned referrals documenting follow-up from an exam or made an appointment with the enhanced protocol vs 20% (20/100) in the standard protocol	54% returned eye specialist report or has appointment (n=47/87)
Noma et al., 2012	Brazil, Guarulhos, Sao Paulo	51,509 screened, 14,651 referred, 5,968 invited for second round of ophthalmology eye exams	1) Financial constraints 2) Weather changes 3) Child or family member sick 4) Logistical challenges, including transportation challenges, scheduling challenges with school, finding someone to stay with younger children, challenges with work schedules, and absent caregiver 5) Lack of confidence in program 6) Parents do not believe screening results 7) Lack of awareness 8) Fear 9) Caregiver forgetfulness	59% (n=8,683/14,651)	A second round of ophthalmology eye exams for children with abnormal vision screening test who missed a first round.	37% (2,228/5,968) attended second round of ophthalmology eye exams [compared to 59% (8,683/14,651) at first round]. Second round resulted in coverage increment of 15% (59 to 75%)	75% (n=10,911/14,651)
Rodriguez et al., 2018	USA, San Jose, California	6,067 screened in demonstration schools, 7,014 students screened in comparison schools between 2007 and 2012	NA	67%	Full-time school nurses at demonstration schools made additional efforts to contact parents (3 times on average) to adhere to referrals to eye care professionals for eye exams, compared to part-time nurses at comparison schools	Follow-up rates in demonstration schools ranged between 96% (2011–12) and 98% (2010–11) vs 69% in the pre-intervention year (2007–2008). Follow-up rates were between 41% and 67% in comparison schools	96%
Suchoff and Mozlin, 1991	USA, Bronx, New York	37 identified in screening as priority cases (hyperopia, myopia or astigmatism ≥ 2D)	NA	NA	1) Hired paraprofessional called parents of priority students on phone 2) If parents could not be reached, a family worker visited the home in the evening 3) Appointments were made for parents to speak to the optometrist at school to emphasize the importance of addressing their child’s visual problems	50% (7/14) attended the scheduled appointment with an optometrist	50% (n=7/14)
Yawn et al., 1996	USA, Rochester, Olmstead County, Minnesota	2,887 screened, 820 with an abnormal vision screening test	NA	NA	1) Multiple referral cards sent home and parents asked to sign referral cards to acknowledge receipt 2) Public health nurses made telephone calls and home visits to families not returning the cards to ensure that each card was received	80% (657/820) had records of optometric or ophthalmologic evaluation after referral. Another 11% (91/820) were wearing lenses for the first time on a subsequent school vision screening.	91% (n=748/820)
Zeng et al., 2020	China, Yudu county, Jiangxi province	9,936 screened (5,053 in intervention and 4,883, in control), 1,114 with abnormal screening results (513 intervention and 601 control)	1) Lack of awareness about eye disease 2) Longer travel time to the hospital and lack of access	(n=225/601)	1) Pre-arranged appointments at local hospital for follow-up care were made 2) Parents were reassured that doctors at the referral center had received professional training	Intervention children had significantly higher compliance than controls (60% [308/513] vs.37% [n=225/601], P<0.001)	60% (n=308/513)
Zhang et al., 2021	China, Shaanxi and Gansu Provinces	15,763 screened, 5,361 had visual impairment, 5,163 were referred	NA	18% (n=962/5,361)	1) Community-based vision center that provides free eye exams and free spectacles following abnormal vision screening 2) Follow-up phone calls to schools and families to encourage parents to bring their children for vision care services	Visitation rate for referred students was 43% (2,237/5,163)	43% (n=2,237/5,163)

ABBREVIATIONS

ECP = eye care professional

WOW = Wills on Wheels, a mobile unit that provides free ophthalmology care at school to low income children with an abnormal vision screenings following optometric exams through Eagles Eye Mobile

KVLF = Kids Vision for Life Foundation

SVP = School Vision Program

US: United States of America; NA: Not Applicable – study did not provide

**Table 3:** Synthesis of Strategies to Improve Adherence to Eye Care Referrals for Children following Abnormal Vision Screening Tests

Intervention Theme	Interventions Identified	Studies Adopting This Intervention
Communication	Phone calls to notify and encourage follow-up eye exams*	(14), (15), (21), (22), (24), (25), (26), (28), (29)
	Parent resources to assist follow-up (list of pediatric eye care providers, their health insurance, and other low-cost/no-cost vision care options) *	(14), (21)
	Assistance with scheduling and coordinating follow-up appointments including pre-arranged appointments*	(15), (18), (24), (30), (27)
	Multiple contact numbers collected at screening for follow-up	(30)
	Detailed informative letter for parents by mail	(22)
	Caregiver- optometrist interaction at school*	(25)
	Referral letters encourage parents and family physicians to arrange exams and forms for eye care professional requesting replies*	(19)
	Home visits to inform parents about abnormal test, if the parent is not available by phone	(25)
Information and Resources	Caregiver resources to assist follow-up (list of pediatric eye care providers, their health insurance, and other low-cost/no-cost vision care options) *	(14), (21)
	Appointments scheduled for caregivers to meet optometrists at school*	(25)
	Developed health education materials for families	(20)
	Caregiver reassurance of the provider training	(27)
Financial Support	Free comprehensive eye exams and prescription glasses within proximity of vision screening location*	(13), (28), (16)
	Caregiver resources to assist follow-up (health insurance accepted, and other low-cost/no-cost vision care options) *	(14), (21)
	Social workers assist with enrollment in relevant health insurance	(30), (20)
	Transportation support – vouchers *	(30), (20)
	Free movie tickets to incentivize follow-up eye exams	(18)
	Financial subsidies for glasses and treatment	(20)
Logistical Support	Free comprehensive eye exams and prescription glasses within proximity of vision screening location*	(13), (16), (28), (20)
	Assistance with scheduling and coordinating follow-up appointments including pre-arranged appointments*	(15), (18), (24), (30), (27)
	Transportation support – vouchers *	(30), (20)
	Reserved slots to expedite referral appointments	(10)

* Stars represent interventions that were represented in more than one theme.

## Data Availability

Data sharing is not applicable to this article as no datasets were generated or analyzed during the current study.

## Abbreviations

US: United States
JBI: Joanna Briggs Institute
ECP: Eye care professional
WOW: Wills on Wheels
KVLF: Kids Vision for Life Foundation
SVP: School Vision Program
IRR: Inter-rater reliability
